# Hip Adductor Muscle Abscess Descending From Septic Symphysitis

**DOI:** 10.7759/cureus.21138

**Published:** 2022-01-11

**Authors:** Benjamin Kraler, Eldaras Gotovski-Getman, Henk Eijer

**Affiliations:** 1 Orthopaedics and Traumatology, Emmental Hospital, Burgdorf, CHE

**Keywords:** symphysis pubis, symphysitis, staphylococcus aureus bacteremia, adductor muscle abscess, pubic osteomyelitis, septic symphysitis

## Abstract

Hip adductor muscle abscesses that descend from an infected symphysis pubis are rare but cause serious morbidity. We present a case of a 73-year-old male patient with unilateral hip adductor muscle abscess that descended from septic symphysitis caused by *Staphylococcus aureus*. Surgical debridement of the adductor compartment could not clear the infection and secondary debridement of the symphysis was necessary to eradicate *S. aureus*. Additionally, we review another four cases with similarities to our case comparing their investigation, treatment, and outcome.

## Introduction

Hip adductor muscle abscesses that descend from an infected symphysis are rare but cause serious illness in the affected individual. In a comprehensive review of septic symphysitis including 100 cases, only one patient was diagnosed with concomitant hip adductor muscle abscess [1]. Nonetheless, awareness of the disease is important because the treatment has to be initiated early and address the adductor compartment as well as the symphysis. Differential diagnoses include septic hip arthritis and the non-infectious condition of osteitis pubis frequently seen in athletes [2]. We report a case of hip adductor muscle abscess secondary to septic symphysitis and present a review of four other cases that were identified in PubMed (US national library of medicine) [3-6]. We discuss their laboratory, radiology, microbiology, treatment, and outcome. Written informed consent was obtained for the publication of this case report and accompanying images.

## Case presentation

**Figure 1:**
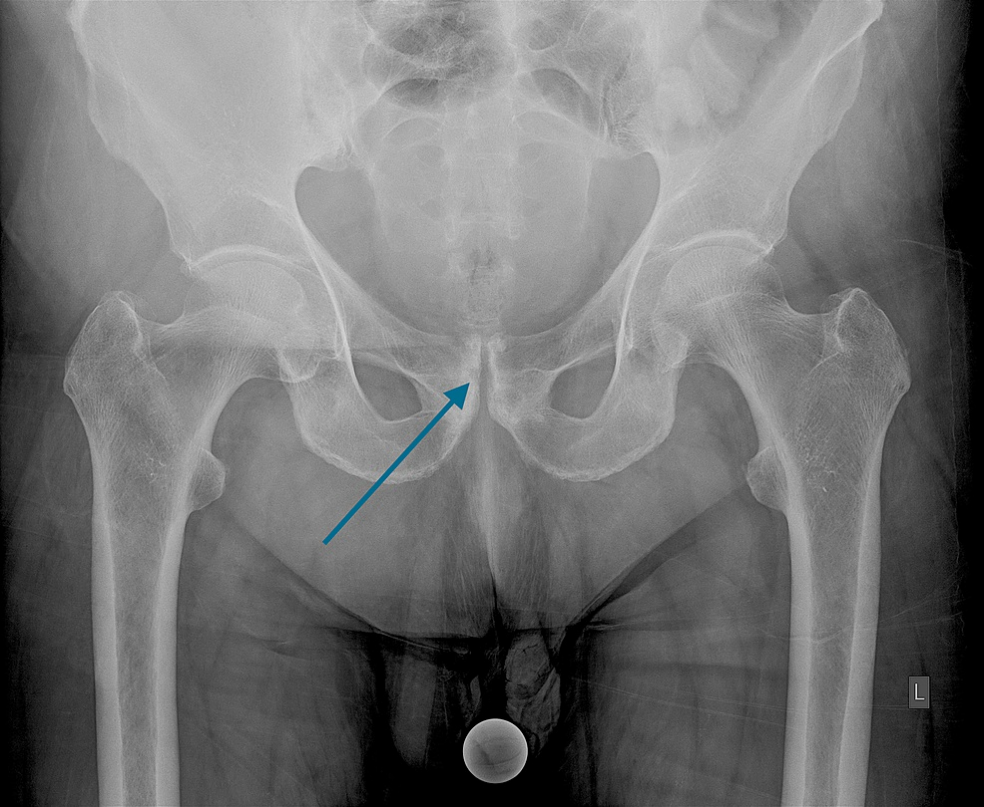
Anteroposterior radiograph of the pelvis at presentation with subtle erosive changes of the symphysis (arrow).

A 73-year-old male patient with a history of sigma resection for colorectal cancer 16 years ago and diabetes presented at the emergency department with acute right groin pain without previous trauma. Vital signs showed a blood pressure of 138 over 65 mmHg, heart rate of 73 bpm, and body temperature of 37.2°C. Local examination of the right adductors showed no swelling or rubor, but tenderness over the proximal adductor compartment. Right hip flexion/extension was limited to 30/0/10 degrees and abduction was restricted to 10 degrees. Pelvic X-ray revealed subtle erosive changes of the symphysis (Figure 1).

The patient was afebrile, but C-reactive protein (CRP) was 149 mg/l with a normal white blood cell (WBC) count. Adductor strain was presumed and he was discharged with analgesic treatment and crutches. Two days later he came back with increasing adductor pain. Laboratory revealed a CRP of 298 mg/l, WBC count of 14.7 × 10^9^/l, blood glucose levels of 8.46 mmol/l, and creatinine of 183 μmol/l. More laboratory results are summarized in Table 1.

**Table 1:** Laboratory results at the time of hospital admission. ASAT: aspartate aminotransferase; ALAT: alanine aminotransferase; y-GT: y-glutamyl- transpeptidase; LDH: lactic dehydrogenase; CK: creatine kinase; CRP: C-reactive protein *Value not within the normal range

Laboratory value (reference range)	Patient's results
ASAT (0-50 U/l)	21
ALAT (0-50 U/l)	18
Alkaline phosphatase (40-130 U/l)	78
y-GT (<60 U/l)	33
LDH (0-250 U/l)	206
CK (0-190 U/l)	208*
Glucose (4.11-6.05 mmol/l)	8,46*
Creatinine (59-104 μmol/l)	183*
Albumin (35-52 g/l)	34*
CRP (0.0-5.0 mg/l)	298*
Sodium (136-145 mmol/l)	138
Potassium (3.4-5.1 mmol/l)	4,3
Calcium (2.20-2.55 mmol/l)	2,21

**Figure 2:**
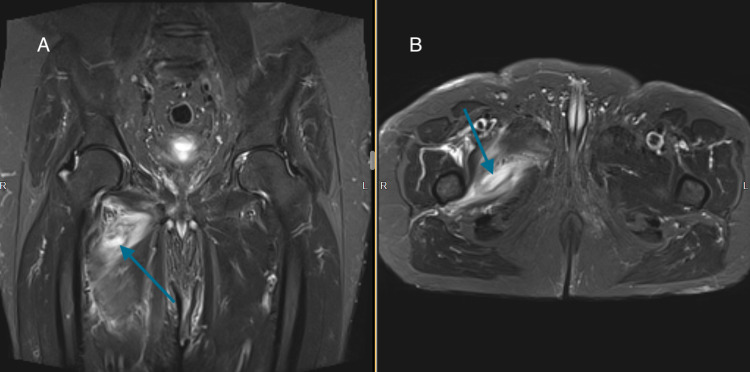
Gadolinium-enhanced T2 turbo inversion recovery magnitude (TIRM) MRI (A) coronal and (B) axial view with hip adductor muscle abscess on the right side (arrows).

**Figure 3:**
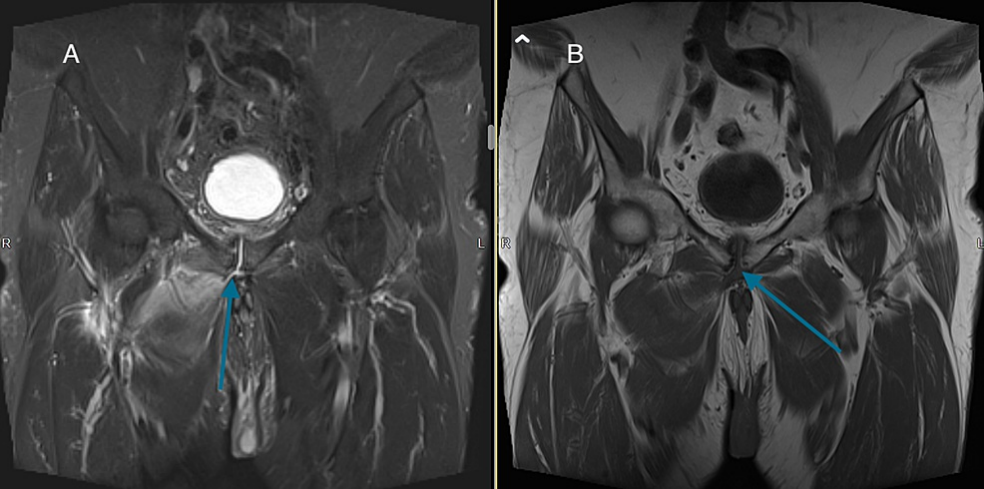
Coronal views: (A) turbo inversion recovery magnitude (TIRM) and (B) T1-weighted image with fluid descending from the symphysis to the right adductor compartment (arrows).

 He was admitted to hospital and pelvic MRI was performed. MRI showed a fluid collection in the right adductor brevis, pectineus and obturator externus muscles (Figure 2). Moreover, fluid in the symphysis and communication of the adductor abscess with the symphysis were identified (Figure 3).

Open debridement of the adductors through a medial approach drained pus. Biopsies and blood cultures identified *Staphylococcus aureus* (methicillin-sensitive). Intravenous antibiotic treatment was started with amoxicillin-clavulanic acid 2200 mg followed one day later by intravenous flucloxacillin 2000 mg four times a day. Blood cultures taken 3 and 5 days postoperatively remained positive, which prompted a follow-up MRI that showed residual fluid collection within the right adductor compartment. Revision adductor debridement and additional debridement of the symphysis through a Pfannenstiel incision were performed. Biopsies identified *S. aureus* at the symphysis and adductors. Due to persisting wound drainage, a third look surgery of the adductor compartment followed 2 weeks later and subcutaneous seroma was drained. Four weeks after the first surgery, CRP was 23 mg/l, WBC count was normal, the wound was bland and the patient was able to ambulate with crutches. Before leaving the hospital the visual analog scale for pain was zero at rest and also when walking with crutches. He was discharged home with a 9-week course of oral clindamycin 600 mg three times daily. At four months follow-up there was no evidence of infection locally or systemically with a normal CRP level. There was no pain over the symphysis or the right adductor compartment and the patient used a cane but was able to walk without aid.

## Discussion

We identified four cases of septic symphysitis with descending adductor muscle abscesses in PubMED that were supported by MRI or CT imaging and had microbiologic confirmation (table 2). Including our case, three out of five patients with adductor muscle abscesses and symphysitis had a surgically treated urologic or abdominal malignancy. Possible other risk factors included diabetes, drug-induced immunosuppression and preexisting osteitis pubis. Although osteitis pubis is an aseptic condition, some authors suggest that the susceptibility to develop septic symphysitis is increased due to degenerative changes of the symphysis [2,7]. Complaints at admission include groin pain, pubic, thigh and/or gluteal pain as well as painful gait. Septic hip arthritis can mimic similar symptoms and needs to be excluded. Fever was present in four and CRP was elevated in all cases with documented CRP levels. Although CRP is unspecific, a normal CRP level makes hip adductor muscle abscesses or septic symphysitis highly unlikely.

**Table 2:** Age, sex, risk factors, complaints, laboratory results, imaging modality, and imaging findings in septic symphysitis with adductor muscle abscess

Author	Age (in years)	Sex	Risk factors	Complaints	Laboratory (WBC in 10^9^/l, CRP in mg/l)	Imaging modality	Imaging findings
Alqahtani et al. [3]	17	M	Juvenile idiopathic arthritis under methotrexate	Groin pain, painful gait, fever	WBC 19.1, CRP 232	MRI	Fluid collection in symphysis and bilateral adductor compartments
Cardoso et al. [4]	57	F	osteitis pubis	Pubic pain, fever	- -	MRI	Fluid collection in symphysis and right adductor compartment
Degheili et al. [5]	68	M	Diabetes, radical prostatectomy for prostate cancer	Pubic pain, painful gait, fever	WBC 11.6, CRP 179	MRI	Symphyseal erosion, pubic bone enhancement, and fluid collection in bilateral adductor compartments
Trubiano et al. [6]	78	M	Transurethral resection for prostate cancer	Thigh, groin, gluteal pain, afebrile	CRP 83	X-ray pelvis, CT, CT-cystogram	X-ray: unremarkable; CT: symphyseal erosion, fluid collection in bilateral adductor compartments; CT-cystogram: cysto-symphyseal-adductor fistulas
Current case	73	M	Diabetes, sigma resection for colorectal cancer	Groin and pubic pain, painful gait, fever	WBC 14.7, CRP 298	X-ray pelvis, MRI	X-ray: symphyseal erosion; MRI: fluid collection in symphysis and right adductor compartment

MRI with gadolinium contrast detected symphyseal and adductor fluid collections in four patients. Interestingly, in one case CT was the imaging modality of choice and CT-cystogram could confirm fistulas from the bladder to the symphysis and bilateral adductor compartments. However, MRI has shown the highest sensitivity and specificity to highlight symphyseal fluid and pubic bone marrow edema in adjacent osteomyelitis as well as adductor muscle abscesses [8]. Pelvic x-ray can delineate symphyseal erosive changes, but it cannot help discern between early stage septic symphysitis and osteitis pubis since both can show symphyseal erosions [2]. Microbiology was secured by adductor and/or symphyseal biopsies or blood cultures and pathogens isolated are summarized in table 3. As one would expect, pathogens found in hip adductor muscle abscesses resemble the spectrum of pathogens in septic symphysitis [1,4-6] with *Staphylococcus aureus* being the most frequently identified pathogen.

**Table 3:** Microbiology, pathogen, management, antimicrobial treatment, follow-up, and outcome in septic symphysitis with adductor muscle abscess

Author	Microbiology	Pathogen	Management	Antimicrobial agent (duration in weeks)	Follow-up (months)	Outcome
Alqahtani et al. [3]	Biopsy adductors	Streptococcus group A	Open debridement adductors	ceftriaxone (6)	6	No infectious sequelae at follow-up
Cardoso et al. [4]	Blood cultures	Staphylococcus aureus	Percutaneous adductor drainage	vancomycin (8)	36	No infectious sequelae at follow-up
Degheili et al. [5]	Aspiration adductors CT guided	Enterococcus spp.	Open debridement symphysis, percutaneous adductor drainage	Vancomycin + meropenem (1) , piperacillin/tazobactam (2) + vancomycin (1.5), rifampicin + ciprofloxacin (8)	6	No infectious sequelae at follow-up
Trubiano et al. [6]	Biopsy symphysis	Candida albicans + Pseudomonas aeruginosa	Open debridement symphysis, cystoprostatectomy	Agent not specified, intravenous (6) and oral (12)	3	No infectious sequelae at follow-up
Current case	Blood cultures, biopsy adductors, biopsy symphysis	Staphylococcus aureus	Open debridement adductors open debridement symphysis	Amoxicillin/clavulanic acid (0.14), flucloxacillin (3), amoxicillin/clavulanic acid (1), clindamycin (9)	4	No infectious sequelae at follow-up

Management of hip adductor muscle abscesses included percutaneous adductor drainage and open debridement of the adductor compartment in two cases, respectively. Primary open debridement of the symphysis was performed in two cases; one was combined with percutaneous adductor drainage [5], the other with cystoprostatectomy due to fistulas from the bladder to the symphysis and adductors [6]. In our case, open drainage of the adductor muscle abscess and antibiotic therapy could not clear the infection and symphyseal debridement revealed *S. aureus* confirming that the symphysis served as the source of infection. In contrast to our experience, Cardoso et al. reported a case of a 57-year-old with symphysitis and an adductor abscess caused by *S. aureus* that healed without surgically addressing the symphysis and by only draining the adductor abscess [4]. However, this was an otherwise healthy individual. In contrast, our patient had undergone sigma resection for colorectal cancer, although this was performed 16 years ago. Likewise, in a 17-year-old with bilateral adductor abscesses following septic symphysitis, *Streptococcus group A* infection was cleared by open debridement of both adductor compartments as a single procedure [3]. This patient received ceftriaxone for 6 weeks and apart from juvenile idiopathic arthritis did not show other comorbidities. This suggests that patients without comorbidities might be best treated by open or percutaneous drainage of adductor muscle abscesses. In patients with medical comorbidities, especially previous abdominal or urologic malignancy, draining the adductor muscle abscess but also debriding the symphysis seems necessary to rapidly clear infection and prevent relapse. Despite different treatment modalities, all patients were infection-free at the final follow-up (range: 3 months to 3 years).

## Conclusions

Hip adductor muscle abscesses originating from septic symphysitis are a rare entity but contribute to significant patient morbidity. Pelvic MRI with gadolinium contrast has the highest sensitivity and specificity to diagnose not only adductor muscle abscesses but also septic symphysitis and fluid communicating between the symphysis and adductor compartment. Microbiologic confirmation is best achieved by tissue samples of the adductors and symphysis. If the patient’s status necessitates immediate empiric antibiotic treatment, at least blood samples for culture need to be obtained before antibiotic therapy is started. *S. aureus* is the most frequently isolated pathogen in septic symphysitis with adductor muscle abscesses but polymicrobial infections can occur. Open or percutaneous adductor drainage in combination with tailored antibiotic treatment seems adequate care in otherwise healthy individuals. However, in patients with previous abdominal or urologic malignancies draining the adductor compartment alone might not be sufficient and debridement of the symphysis is needed to clear the source of infection. Serial CRP and repeat blood cultures help to monitor the success of surgical and antibiotic treatment.
